# On the Diseases of Women; Including Those of Pregnancy and Childbed

**Published:** 1857-08

**Authors:** 


ART. VII.—*On the Diseases of Women; including those of Pregnancy and Childbed.* By FLEETWOOD CHURCHILL, M. D., T. C. D., M. R. I. A., Vice-President and Fellow of the King and Queen's College of Physicians in Ireland; one of the Presidents of the Obstetrical Society, etc., etc., etc. A new American Edition, Revised by the Author. With Notes and Additions by D. FRANCIS CONDIE, M. D. Philadelphia: BLANCHARD AND LEA. 1857.

This work of Dr. Churchill is an old and familiar friend in a new and greatly improved dress. A succession of editions has attested the general favor with which this work on Female Diseases has been received by the profession. The present edition has, perhaps, superior claims upon the American medical profession, for it has had the personal revision of the author, and he gracefully "Dedicates it to the Members of the Medical Profession in America, as an expression of his admiration for their Intelligence, Energy and Scientific Attainments; and of his gratitude for the kind approbation they have extended to his works."

Besides a general and elaborate revision, which includes numerous additions, three entire chapters have been added on tetanus, paralysis, and arterial obstruction, as they occur during gestation or in the parturient female. Additions have also been made by the American Editor, Dr. Condie;—for it would seem that as a matter of necessity every foreign author must have an editor to give him credence, or to stand sponsor for, or to soften and modify the doctrines taught, for fear the pure and unadulterated original would be of difficult interpretation, or lead to serious errors in practice.

A review in the ordinary acceptation of the term, is not to be expected of a work which has been so long before the public, and been so frequently the subject of critical examination and notice. We shall not even attempt to designate the numerous subjects treated in the volume, for our space would but render any such allusion little better than the page of an ordinary index. We design to notice only, the prominent additions made to this volume which mark it particularly in contrast with previous editions, and such other subjects as may from their prominence claim for them more particular notice. It is sufficient to say that the work covers very completely the whole of the ground embraced in that multitude of ills known as "female diseases," and the different subjects appear to be treated as fully as is possible for them to be in the compass of a volume. The authorities quoted are carefully acknowledged in notes, and the references are copious, showing the amount of labor bestowed upon the preparation of the volume. Such a work of course does not necessarily display any very great amount of originality in the author, — the honors of the discoverer are the rewards of but a very few of all those who really toil, — but he is most certainly not undeserving of commendation who so successfully tills the soil of medical literature as to be capable of separating the tares from the real grain, and presenting to his brethren in one sheaf all that is valuable, from the seed broadcast through a long succession of years, and from the hands of a multitude of sowers. Indeed, to the credit of Dr. Churchill for honesty, he has never claimed originality for his work, for in the preface to the first edition he says, "it will be evident that the volume has no higher pretensions than that of being a compilation, with the addition of whatever information I may have acquired from hospital or dispensary practice. I have endeavored to ascribe each opinion to its true author, and to appropriate none that are not strictly my own." The present edition comes to us, "with a sincere hope that we will find it improved. Time has given him more experience, and the records of science show that others have not been idle. He has most carefully revised the work, pruning what appeared to be exuberant; enlarging what appeared defective; and adding the results of others' experience and his own."

To ourselves one of the chief merits of the volume is the excellent order and system of classification which is observed throughout the volume. There is no difficulty in turning at any moment, to any subject upon which it treats. Commencing with the diseases of the *external* organs, the work systematically pursues its course through the consideration of all the diseases to which the female genital system is obnoxious, either in its virgin state, or in the changes wrought during gestation, or the nearly innumerable sequalæ which follow too often in the train of the crowning effort of the uterus, parturition.

In conformity to the intention which we have already intimated, we shall first proceed to notice the three chapters which are newly added to the present edition, and after that follow the subjects as far as time, and our limited space will allow.

*Tetanus*,—Dr. Churchill observes, although a very fatal affection, has been considered so rare an attendant upon childbed that it is scarcely noticed by any writer upon diseases of women. Dr. Simpson has, however, collected a sufficient number of cases to prove that it should no longer be overlooked, and a paper by him forms the basis of the article in the volume before us.

Traumatic tetanus as proved by statistics, and in opposition to the former general belief, is much more frequent in the male than in the female. Dr. C. gives statistics, principally European we presume, which rates the proportion nearly 4 males to 1 female.

This ratio is, however, much higher than we find it in this country. The U. S. mortality statistics for 1850, give 694 deaths by tetanus for the year, of which 418 were males and 276 females, which reduces the comparative liability of the males to less than 2 to 1. We apprehend that few persons are aware that the mortality from tetanus in the United States, reaches so high a figure as 694 deaths in the year, as is given in the above returns. In this aggregate is included the number of 416 blacks. We may, perhaps, add to the value of these figures by specifying the proportion of the sexes in both races. Among the white population, the deaths by tetanus were, males 181; females 97. Colored, males 237; females 179. The season of the year appears to have but little influence upon its production. Figures never more plainly exhibited a fact, than the uniformity with which the disease is equally distributed throughout the year.

Tetanus may occur from injury of the *unimpregnated* uterus, although this is very rare.

Professor Simpson gives a case in which he removed a large cellular polypus "by slight traction" from the uterus, and on the ninth evening lock-jaw came on, and terminated fatally in about fifty-five hours.

"It may also occur *after abortion*, as was held by very ancient authorities; but it is not peculiar to first pregnancies, or to any period of pregnancy. Nor was there any regularity in the period which elapsed between the miscarriage and the sitting in of tetanus. In one it occurred a few days after; in another on the sixth day; in a third on the seventh; in a fourth on the eighth; in a fifth on the thirteenth day, and in a sixth a fortnight after the miscarriage. All the patients died; one in sixty hours; one in seventy hours; three on the third day; one on the fourth day; one on the seventh day; one on the eleventh day, and one on the thirteenth day."

There was nothing peculiar about the miscarriage in most of the cases; in some the ovum was not immediately thrown of; and in others there was so much hæmorrhage as to require the plug. But irritation from retention of the ovum, or the presence of the tampon is an every day occurrence without exciting tetanus.

"The symptoms of tetanus were in no respect unusual—commencing with a degree of stiffness about the jaws, and they shortly became rigid and the body retroflexed by tetanic spasms."

The lesions discoverable in the uterus after death, in the cases following abortion, shed no light upon the character of the injuries and their connection with the development of fatal disease.

Tetanus may also occur after *parturition.* Dr. Churchill speaks of the collection of 250 cases, following labor.

Our author says, "It is not more frequent after the first confinement than after subsequent ones; but as to the period of the attack, Dr. Simpson observes, that it seems to be governed 'by the same laws as regulate the occurrence of the disease after abortion, or after surgical operations and injuries. Under all these conditions the tetanic attack usually does not commence till about a week after the occurrence of the exciting obstetrical or surgical lesion. According to some statistics published by Romberg, in more than a half of all the instances of surgical tetanus—or in 112 out of 208 cases—collected by him, the attacks set in between the third and tenth days after the receipt of the injury, or the occurrence of the operation.' Of Dr. Simpson's cases, it is mentioned, that in one it occurred soon after delivery; in one on the second day; in one on the third; in one on the fourth; in four on the fifth; in one on the sixth; in one on the seventh; in two on the fourteenth; in one on the seventeenth day; in one after three or four weeks; and in one after seven weeks. In Dr. Woodhouse's case it occurred on the eleventh day. Of Mr. Waring's 232 cases, seven were attacked the first day; thirty-two the second; twenty-nine the third; twenty-three the fourth; twenty-two the fifth; thirty-two the sixth; fifteen the seventh; fourteen the eighth; fifteen the ninth; fourteen the tenth; two the eleventh; nine the twelfth; four the thirteenth; one the fourteenth; one the seventeenth; one the eighteenth. In Dr. Patterson's case it occurred on the fourteenth day."

There is nothing peculiar in the character of the tetanic symptoms, except that early in the attack it may be mistaken for a species of sore throat, and our attention diverted from the prompt treatment of a very severe affection. The disease seems more frequent in warm climates than in cold; Mr. Waring has published an account of the disease occurring in puerperal women at Bombay, from which it appears that in three years, ending Dec., 1852, no less than 232 women died from it, and the number appears increasing.

Cold, injuries, operations, and, perhaps, the presence of putrifying matter in the uterus, are assigned as the principal exciting causes. As to the pathological character of the disease, Dr. Simpson attributes it to the lesion of the internal surface of the uterus, which he considers analagous in its operation to the external wounds, producing tetanus, and the reason why it does not more frequently cause it, may be from the uterus being almost entirely supplied with nerves from the sympathetic system.

Upon the subject of treatment, after detailing the course usually employed for the relief tetanus, the use of chloroform is recommended. If used, its action requires to be sustained for many hours, or oftener, perhaps, for many days. In illustration, a case is given where its beneficial effects were apparent; but the report looses great part of its value as it fails to state the actual termination, the reporter ending the recital by saying, "I now anticipate a recovery."

*Paralysis occurring during Gestation and in Childbed*, forms the second chapter of the important additions to the present volume.

Dr. Churchill states, that having been much interested in a case of paralysis after delivery, he was induced to enter into the inquiry of the occurrence of this disease, not merely after delivery, but during gestation, and to examine all the authorities within his reach for information relative to the same, but his search had not been very fruitful of results. In the principal obstetrical writers no mention is made of the disease.

Reference is made to all the authors, and reporters of cases in periodicals, which a patient search discovered, who speak of this complication of the puerperal state, which renders the chapter a very complete bibliographical notice of this form of palsy.

A communication by Dr. Simpson, made to the Edinburgh Obstetrical Society, is quoted, where the connection between the presence of albuminuria and the development of the puerperal convulsions and paralysis, is traced. The derangements of the nervous system assume a variety of forms, and may even be developed, without preceding convulsions. Some form of blood poison seems to be at the origin of these difficulties.

We quote the following paragraphs, in which Dr. Churchill sums up what is known upon the subject:

"Let us now see to what the information we have obtained from these different authorities amounts. Very briefly, we find:

"1. That hemiplegia, paraplegia, or partial paralysis, may occur previous to, during, or some time after labor.

"2. That by some authors, the paralysis, in paraplegia especially, is attributed to pressure upon the muscles or nerves, in prolonged labor; but this is also denied, as the same disease follows easy labor, or occurs after the lapse of some days.

"3. Paralysis may terminate convulsions or accompany them.

"4. Paralysis may be the consequence of organic disease, or of effusion into or upon the brain or spinal marrow.

"5. Paralysis may result from reflex action.

"6. The palsy may depend upon temporary causes, and among such causes albuminuria may be included.

"7. Hemiplegia may run on into apoplexy, or it may pass off in a few weeks, or sometimes more slowly. Paraplegia may leave a temporary or more permanent lameness; the local palsies (amaurosis, deafness, &c.) generally last but a moderate time.

"8. A nervous or hysterical paralysis, may occur occasionally in the unimpregnated state, or during pregnancy, but that it seldom continues after delivery."

The above summary of pathological points, is followed by the recitation of thirty-five cases, in which all the numerous forms of puerperal paralysis is described. We have hemiplegia, paraplegia, paralysis of arms, hands, and legs, deafness, facial par alysis, amaurosis, partial paralysis of side, etc., occurring during pregnancy, and during and after delivery, detailed in all their various phases. Of the thirty-five cases, twenty-three occurred during pregnancy; twelve either during or after labor. Of the thirty-five cases, four died.

In most of the cases, the attack was without warning, and without *apparent cause*. And it is a difficult matter to assign a cause. Our author enters into a careful examination of the various causes which have been assigned in the morbid states thus developed in the nervous system, but arrives at nothing definite. The most constant abnormal condition of the system noticeable, is the presence of albuminous urine. Albuminuria is a very general, although not constant accompaniment, of puerperal convulsions, whether paralysis follows or not the eclampsia. And albuminuria is present in these cases of paralysis, without any reference to their being preceded or not, by convulsions. And hence it follows, according to Dr. Lever, that as the albumen diminishes, the paralysis subsides. In view of all the circumstances attendant upon this consideration of the subject, Dr. Churchill concludes it is probable the kidneys play a more important part in these paralytic affections than has been suspected, and that the subject deserves more attention than it has received.

"Nor is this barren theory only," adds Dr. C., "but, if it be true, it has a direct bearing upon practice, inasmuch as our attention ought not to be confined to the secondary affection of the nervous system, in such cases, but must be directed to the relief of the renal malady, and to the restoration of the kidneys to such a state of efficiency as may enable them to remove the morbid constituents of the blood; and for our encouragement, we have seen that a diminution of albumen in the urine is followed by mitigation and cure of the paralysis."

With this view, bloodletting general, or local as the system will bear; blisters, purgatives and mercury, are the remedies proposed, their use being modified according to the condition of the patient, and the circumstances of the attack. In most of the cases given, and to which we have already alluded, mercurials were given to the production of salivation, more or less complete.

*Arterial Obstruction in Puerperal Women*, forms the third chapter of additions to the present volume. It is a short and very concise account of this affection, in which the symptoms are principally given by the recitation of cases. The chapter is one which is so thoroughly condensed by its author, that its entire transferrence would alone enable us to give in this place any adequate idea of its contents.

*Convulsions*, follow undoubtedly, the most regularly in the order of consideration, after the diseases to which we have just made reference. In the volume before us it occupies the sixteenth chapter of the third book. Puerperal convulsions, Dr. Churchill divides into three varieties—the *hysteric*, the *epileptic*, and the *apoplectic*. He enters minutely into the diagnostic symptoms of these varieties, so much so that one would naturally conclude that there is but little difficulty in, at all times, specifically diagnosing and describing all the shades of differences which may present themselves at the bedside. We cannot divest ourselves of the belief, that the various diagnostic symptoms are more easily described upon paper, than they are made out in the sick chamber, notwithstanding all the acumen the practitioner may bring to bear upon the investigation of the case. The hysteric form may present but few difficulties, but after all, we are inclined to believe that *a*, or *the differential* diagnosis is oftener made out from the *result* of the case, than from discoverable differences in the symptoms apparent during the continuance of the attack.

Epileptic convulsions are said to be far more frequent than either of the other forms. Apoplectic convulsions are said, seldom or never to occur, except toward the termination or after the conclusion of labor.

The statistics are presented in the volume before us, of 190,313 cases of labor, in which convulsions occurred 273 times, or 1 case in about 693$\frac{3}{4}$.

The mortality is large, though probably less now than formerly. Thus, out of 254 cases, given in the volume, 57 mothers were lost, or about 1 in 4½. Of 15 cases treated by Dr. Condie, 3 of the patients were lost. We must admit, that this is a far more favorable rate of mortality than we had been led to suppose.

We shall make no attempt at a recitation of the numerous *causes* which have been assigned and are noticed, for the development of puerperal convulsions; these, of course, must be more or less hypothetical, and be the result of theoretical reasoning, rather than the conclusions drawn from positive demonstration. We shall stop only to notice, amid the numerous disturbing causes which have been assigned, the prominence which is given to the dependence of convulsions upon an albuminous condition of the urine. The authorities quoted go to prove, that in a large proportion of cases of convulsions there is albuminuria, with or without anasarca. Still, albuminuria may be present without convulsions, and convulsions may supervene without albuminuria.

"For example, Dr. Blot found albumen in the urine of 41 pregnant women out of 205, and chiefly in primiparæ; and Dr. Litzmann examined the urine of 131 females, 79 during pregnancy, 80 during labor, and 80 after delivery; albumen was present in 37, and absent in 95; of the 37, 26 were primiparæ. What is the exact relation between the two, it is difficult to say precisely. I believe, with Dr. Simpson, that they both stand in the relation of effects of another cause, viz: 'a pathological state of the blood, to the occurrence of which pregnancy some way disposes.'"

"More recent researches have thrown a good deal of light upon the occurrence of this renal affection. De Villiers and Regnault observed it as early as the sixth month; Litzmann not till the eighth. The most characteristic symptom is dropsy of the hands, arms, and face, but dropsy does not necessarily coëxist."

To congestion of the kidney produced by the mechanical pressure of that organ by the enlarged uterus, has the presence of albuminuria been attributed, rather than to the consequence of granular degeneration. According to most observers, the albumen very shortly disappears, often as early as forty-eight hours after delivery.

Pregnant women are more especially liable to puerperal convulsions during the latter two months of pregnancy; this is not, however, invariable, for they may appear at an earlier period, and at irregular intervals. The risk of an attack is increased by the approach of the date of the termination of gestation, the extreme and increasing distension of the uterus, with its consequent increased irritability furnish the apparent cause.

The *treatment* of convulsions, under the head of the epileptic form, is upon the whole judicious, and calmly inculcated. He divides the cases into two classes, sthenic and asthenic, and modifies the character of his treatment by the condition of his patients. To the pale, exsanguined and weak he would prescribe the loss of blood with the exercise of great caution, and place his main reliance upon counter-irritation of the head and neck, the application of cold in moderation, and the use of purgatives, and, he "believes, upon anæsthetics." In the sthenic form, with the head hot, face flushed, and pulse full, firm and frequent, he would practice venesection freely and early, and use in connection the other means before indicated.

He speaks in terms of approbation of the use of anæsthetics, and quotes with terms of commendation, authorities and cases where relief has been afforded by producing insensibility by these means.

*Apoplectic convulsions* are treated separately, and the causes, symptoms, pathology, diagnosis, and treatment are all briefly considered. The distinctive characters which the author would inculcate as present in this peculiar form, are sufficiently apparent in the descriptive name attached to it. It is said, seldom or never to occur except toward the termination, or after the conclusion of labor, and as being evidently caused by the stress upon the cerebral vessels occurring during labor.

This form, as might be supposed, is most certainly fatal, but there occurs occasionally, cases answering, the author presumes, to the congestive apoplexy of Abercrombie and Lallemand, where our timely aid is successful, and the patient recovers sense and motion; and if proper care be taken, is speedily well.

An editorial note included in brackets, of a few lines less than a page, concludes the chapter. We must confess that we were surprised to find, that the note thus appearing as Dr. Condie's, has been transferred nearly bodily from one of Dr. Huston's notes to the edition edited by himself, without proper credit to the original annotator. We have before us a copy edited by Dr. R. M. Huston, and published in 1847, in which Dr. H. incorporates a note in that portion of the text devoted to the consideration of the epileptic form of convulsions. Dr. Condie, in the edition under review, transfers this note to the *close of the chapter* on convulsions, and following immediately after the consideration of the apoplectic form. The first paragraph he credits to Dr. Huston, the balance of the note he appropriates as his own. This, with two or three slight and immaterial alterations, is a *verbatim* copy of Dr. Huston's note! which fact any person can verify by making the comparison.

This may be a cheap method of *editing* a book, but we can but consider it a means altogether beneath the dignity of a man with Dr. C.'s reputation. The more so as the note adds not one syllable to the value of the text. Dr. Condie cautions against the excessive use of bloodletting, and speaks favorably of the use of cold applications, purgatives, &c.;—so does Dr. Churchill,—and he does still more, he speaks favorably of and recommends anæsthetics. Dr. Condie preserves a profound silence upon the subject; you would not know from the teachings of Dr. Condie that such remedies had ever been used.

*Puerperal Fever* is treated at length. The author gives some sixty pages and the editor adds some ten or twelve more.

Dr. Churchill omits the detailed historical accounts of the prevalence of the disease at distinct periods, given in the earlier editions, and contents himself with simply presenting a chronological list of the epidemics, and the pathological characteristics when ascertained. Numerous statistics are also presented in other tabular forms.

The subject is treated at too great length for us to give, in our limited space, anything more than a very meagre outline of its contents.

He acknowledges the general fact that puerperal fever is connected oftentimes with the presence of local disease, and repeats as his conviction that there are not many cases of fever without some lesion of the organs employed in parturition, or of the neighboring tissues; but he now abandons the position taken in the former editions, in conformity with the opinion held by Dr. Robert Lee, that there is no specific, essential, or idiopathic fever, which attacks puerperal women, independently of any local affection in the uterine organs. Dr. Lee taught, "that as the constitutional symptoms appear to derive their origin from a local cause, it would certainly be more philosophical and more consistent with the principles of nosological arrangement to banish entirely from medical nomenclature the terms puerperal or childbed fever, and substitute that of uterine inflammation, or inflammation of the uterus and its appendages, in puerperal women."

Dr. Churchill now says, "I am bound to state honestly and frankly that more extended experience has led me to doubt the accuracy of these views, and to believe that malignant puerperal fever is something more than a local affection, and that the constitutional disease is often rather primary than secondary. At the same time I have no doubt that Dr. Lee's views are applicable to many cases."

What is the essential nature of malignant epidemic, puerperal fever, is a question, which he does not find of easy solution. He gives at length the different views held at different times by a multitude of writers.

Dr. Churchill sums up this branch of his subject as follows:

"If we regard the peculiar characteristics of different epidemics, we find them extremely varied. In one, the lochia are suppressed; in another, they are profuse; and in a third, unaltered. Diarrhœa is common in one epidemic, constipation in another; typhoid symptons in one, inflammatory in another. And as to the effects of remedies, we find as great a diversity: one high authority recommends saline purgatives, which fail in the hands of other practitioners; another loses his patients until he bleeds largely at the commencement, whilst others lose all who are so bled. Calomel is the universal remedy in one epidemic, opium in another, purgatives in a third, inunctions in a fourth, turpentine in a fifth, &c.

"Now from these variations, the inference is obvious, that the *type of the disease varies in different epidemics, and that the treatment must necessarily differ*. But I think we may go a step farther; and if any one will carefully compare a case of simple inflammation of the womb or peritoneum in child-bed, with a case of malignant puerperal fever, their symptoms, general and local characteristics, course, and the effects of remedies, they will be obliged to come to the conclusion that, although the latter may exhibit local disease, it is not exclusively nor primarily a local affection."

From what observation we have been able to make upon epidemics in any form, we are inclined to believe, that in the above paragraphs is to be found the key which is to afford a solution to the inconsistencies which are apparent in medical opinions and practice when applied to epidemic diseases from year to year, or through a succession of years. A stereotyped form of practice is not an intelligent exercise of the attainments of the physician, nor all that is to be learned from the teachings derived from the observation and study of the phenomena of disease.

The subject of the *contagious character* of puerperal fever is discussed at considerable length, and the arguments and authorities *pro* and *con* are cited, and Dr. C. comes to the conclusion, "after a close and careful examination of the history of epidemics, of cases recorded, and of the opinions of men of the greatest experience, that the weight of evidence is in favor of puerperal fever being infectious and contagious." He says, however, "Of the simple cases of peritonitis or phlebitis after labor, occurring sporadically, he does not know that any one considers them contagious." He dwells somewhat at length upon the apparent connection of erysipelas and puerperal fever, where these diseases assume an epidemic form, or where erysipelas appears to be the precursor of the latter fatal affection.

Dr. Churchill inculcates great caution, as a matter of necessity to be observed by those, who by any possibility may be the means of propagating this disease. It is most certainly a thought which can add nothing to the happiness of the physician to believe, that when called to prescribe to the sufferings of the distressed, that instead of being the assuager of pain, and the minister of health, his presence is only to certainly sow the seeds of inevitable death.

At this period, Dr. Churchill proceeds to discuss the different forms of disease. He first gives the arrangements or classifications adopted by others, and then presents the arrangement which he proposes to employ in the consideration of the subject.

We have no space or time to follow him through all these differences of classification, as he proceeds to describe the symptoms, diagnosis, prognosis and treatment of each separate form, or variety.

Under the head of *treatment*, we can do but little more than to intimate, that he predicates it "upon general principles," (as we have known many a puzzled student unable to descend to particularities, answer at the "quiz" in the lecture room,) which govern the treatment of local inflammatory disease. Bloodletting, general or local, singly or combined; calomel and opium, &c., are indicated and prescribed. One cannot, however, avoid noticing the freedom from dogmatism which pervades the pages upon the subject of treatment, and the cautions which are inculcated against attempting to treat these various forms of disease by the adoption of one invariable, and inflexible rule. Attention to the peculiar type of the disease as it presents itself to the eyes of the practitioner, and makes its demand upon him at the bedside of the patient, is strongly urged upon the consideration of the physician, and he would indeed be deaf to every call of duty who heeded not the voice of so rational a teacher. Upon the employment of bloodletting, the recommendations are judicious, and no one is taught to sacrifice his patient, at the mere suggestion of the presence of a disease, although it comes in so fatal a form.

We notice in one or two places, he speaks of the use of opium in *large doses*,—*a grain* administered every hour, and with favor. What sort of a dose would Dr. Churchill call *a grain of morphine* given every one or two hours, in the treatment of this disease?

In the additional pages which Dr. Condie has added to this chapter, he endorses generally the positions taken by Dr. Churchill. Indeed he appears to insist more strongly than the latter gentleman, upon the idiopathic character of this form of fever, and can find no other means of explaining the difficulties which are apparent upon any study of the phenomena of the disease, than by a recognition of puerperal fever as a true idiopathic fever.

Dr. Condie says that "Dr. Meigs in his recent treatise on childbed fever, repudiates the existence of an idiopathic puerperal fever, inasmuch as the term fever excites 'a certain material idea of zymosis in the mind of the hearer of it;' but he, nevertheless, substantially admits all that would be necessary to include puerperal fever in the list of zymotic diseases."

Calling into question the position assumed by Dr. Meigs, that the atmospheric causes of this disease do "not poison men, nor boys, or girls, or non-pregnant women, but only the pregnant or lying-in portions of society," Dr. Condie proceeds to trace the parallelism which has so apparently existed between the outbreaks and developments of erysipelas and puerperal fever, which he illustrates by reference to the numerous epidemics which have been rife within the United States within the last few years. While the mother, exhausted and helpless from the pangs of labor, was marked as a special victim, male as well as female, and young as well as aged fell before the disease. Inflammation of the peritoneum in men and nonparturient fe-females, as well as the deadly epidemic erysipelas kept close company with puerperal fever as it enveloped in the shadows of the grave the lying-in-chamber. There was no room to doubt the intimate connection between the two forms of disease.

We notice that neither the author or editor makes any reference to the treatment of puerperal fever by large doses of opiates frequently administered,—we do not mean grain doses of opium, but grain doses of *morphine* administered every one or two hours, assisted by tonics and concentrated nutriment. A number of cases within a few years past, have found their way into the medical periodical press, and they are sufficiently full of promise to challenge the attention of every practitioner.

*Congestion, Inflammation, Erosion and Ulceration of the Cervix Uteri*, are treated of in a chapter of moderate length, and which in our irregular course of exploration of the contents of the volume, we propose next to examine. He purposely, as he declares, avoids entering into the controversy which has been carried on with so much virulance upon this subject, and refers to the writings of the acknowledged champions of the diverse sides.

But he believes most decidedly in the presence of local disease in connection with uterine derangements, and follows Bennett and Whitehead as "the men of his counsel." He is, however, moderate in tone, and avoiding all discussion, describes symptoms and appearances only, not seeming to doubt in the least but what these lesions exist and can be found for the seeking after; and has nothing to say about "dishonesty" of purpose and practice, upon the part of those who may differ with him,. as was the free epithets of a recent writer, whose work we had occasion to refer to not long since.

The symptoms accompanying the several conditions of the os uteri placed descriptively at the head of the chapter are each considered in their order. We can, however, but consider this more as an attempt toward scientific accuracy in treating of his subject, than a belief of its practical value as a means of distinctive diagnosis. For we have no idea that anything else than an examination aided by the speculum, will enable one to decide upon the actual presence of disease and its specific character, although the experienced practitioner in these diseases may shrewdly suspect and predict with great certainty from the symptoms, the great cause of the whole train of ills which has "legion" for its name, and renders woman's life oftentimes a burthen.

Nor do we understand that very much difference in the mode of treatment is inculcated in these several forms; or a very dissimilar result obtained in either case.

But we are unable to pursue the analysis of the volume any further at this time. We think we have already presented enough of its contents to disclose the practical character of the work, and its value to the practitioner as a ready means of reference upon all points presented to his consideration while treating the diseases peculiar to the female.

Dr. Condie has interspersed a number of notes throughout the volume, and they seem to be principally added to those articles which we have not made the subjects of especial notice. Dr. Condie, in these notes, seems to have supplied a number of omissions in the text, necessary to render the subjects complete; and what we notice with especial favor is, his notes have particular reference to the labors and contributions of American medical men in the departments of medicine and surgery.

The labors of both author and editor seem to have been devoted to bringing the work up fully to the times, and we feel that we can endorse the prefatorial note of Dr. Condie, who regards "it a complete and faithful exponent of the present state of medical opinion and experience, in reference to the pathology and therapeutics of the entire range of diseases to which the female sex is liable, including those of pregnancy and childbed."

For sale by PHINNEY & Co. N.

